# The strategic role of human resource managers in shaping decision-making in Ethiopia

**DOI:** 10.1371/journal.pone.0327296

**Published:** 2025-07-08

**Authors:** Tekalign Gidi Kure

**Affiliations:** Department of Management, College of Business and Economics, Wachemo University, Hosaina, Ethiopia; Higher Education Partnership / Erasmus University Rotterdam, ETHIOPIA

## Abstract

This study thoroughly examines the role of human resource managers in shaping decision-making within the manufacturing sector in Ethiopia. Employing a mixed-methods research design, data were gathered from 282 human resource directors using structured questionnaires and supplemented by 24 in-depth interviews. The quantitative analysis involved the use of descriptive statistics, the Kruskal-Wallis test, and the Mann-Whitney U test to explore orientations and differences across sectors, while qualitative data were analyzed using thematic analysis to capture the nuanced perspectives of Human Resource (HR) managers. The findings reveal a widespread recognition of HR as a strategic asset, with the majority of respondents advocating for greater integration of HR into organizational strategic planning. Nevertheless, the study uncovered significant sectoral disparities in the degree of HR managers’ involvement in strategic decision-making. Notably, the textile and clothing industries reported higher levels of HR representation in strategic discussions compared to the chemical and pharmaceutical sectors, where HR’s role was less pronounced. Additionally, the research identifies a critical gap in the formalization of HR strategies, with many organizations lacking documented, comprehensive HR plans. This absence of formalized HR frameworks limits the strategic potential of HR departments, restricting their ability to contribute effectively to organizational performance. The study underscores the critical need for bridging the gap between strategic intent and actionable HR practices is crucial for achieving sustained competitive advantage in the manufacturing sector.

## 1. Introduction

Strategic Human Resource Management (hereafter SHRM) has emerged as a critical framework for aligning Human Resource (HR) practices with overarching business strategies to enhance organizational performance, drive innovation, and foster sustainability [1]. In recent years, SHRM has gained prominence in academia and industry, reflecting a growing recognition of HR’s strategic contributions. Historically viewed as a support function, HR is now seen as integral to achieving competitive advantage, particularly in complex and dynamic environments. Recent studies highlight the essential role of HR in shaping high-level strategic processes, emphasizing the need for HR’s involvement in organizational decision-making [2]. SHRM is considered pivotal in aligning HR practices with organizational objectives, enabling firms to better navigate external challenges such as globalization, technological advancements, and workforce diversity [3–5]. This shift underscores the strategic importance of HR in ensuring that organizations remain competitive and adaptable.

The strategic role of HR has received growing attention in both academic and practical fields, with scholars increasingly focusing on the integration of HR into wider business strategies to strengthen organizational sustainability and resilience. For instance, Božac and Kostelić [6] highlight the crucial role HR managers play in developing and implementing strategies that align human resource capabilities with organizational objectives. Likewise, Hermans and Ulrich [7] emphasize a shift from HR’s traditional administrative duties to a more active role in business strategy formulation, underscoring HR’s influence in shaping strategic decisions and promoting high-performance work practices vital to organizational success. A recent study by Anvari et al. [8] revealed that emotional intelligence has the potential to shape strategic human resource management practitioners into professionals who prioritize people-oriented activities. This expanding literature underscores HR’s growing influence as a strategic partner in organizational achievement.

The recent global crises, such as the COVID-19 pandemic and the surge in digital transformation, have further underscored the strategic importance of HR. Vaiman et al. [9] discuss how HR functions were pivotal in managing workforce resilience, employee engagement, and remote work strategies, all while ensuring organizational continuity during the crisis. Moreover, the significance of digital HR strategies is found in the integration of data-driven decision-making and artificial intelligence, which enhances the efficiency of workforce planning and development [10–12]. These developments illustrate how HR managers must be deeply involved in strategy formulation to mitigate risks and capitalize on new opportunities. SHRM is now critical for addressing broader organizational challenges, including talent shortages, leadership succession, and diversity management, all of which have become central to maintaining competitive advantage in a post-pandemic world [13].

### 1.1. Statement of the problem

In the context of emerging economies like Ethiopia, the relevance of SHRM becomes even more pronounced, given the unique developmental challenges and opportunities present within the industrial sector [14]. The manufacturing industry in Addis Ababa and its surrounding regions plays a crucial role in Ethiopia’s industrialization efforts, significantly contributing to the nation’s economic growth and structural transformation [15]. Despite the strategic importance of manufacturing in Ethiopia’s economic framework, there exists a notable gap in research regarding the application of SHRM within this sector, particularly concerning the involvement of HR departments in strategic decision-making processes.

The implementation of SHRM in Ethiopian manufacturing firms is still in its nascent stages, as many organizations face challenges related to capacity building and skill mismatches, which hinder effective strategic HR practices [14,16–18]. For instance, Tekleselassie et al. [17] noted that low productivity levels in the Textile and Garment industry can be largely attributed to insufficient educational backgrounds among the workforce. Additionally, Hailu and Tanaka [19] pointed out that, despite having abundant human resources, the overall quality of labor in Ethiopia is subpar, mainly due to the absence of effective and systematic training programs aimed at enhancing worker productivity. Moreover, traditional HRM practices, such as local advertisements and word-of-mouth, continue to dominate the manufacturing sector, as reported by Hailu et al., [20]. Eshiete [14] indicated that the reliance on unskilled labor, combined with outdated recruitment methods, limits access to skilled labor, further compounding the challenges faced in the sector [16–18].

Despite these insights, there is a significant gap in examining strategic HRM orientations across broader manufacturing sectors in the country, particularly in larger firms. Previous studies have primarily focused on operational HR practices rather than HR’s strategic role in decision-making. This study aims to fill this gap by exploring how strategic HRM is integrated into decision-making processes in manufacturing firms, using both quantitative and qualitative data from senior HR managers. By doing so, it will contribute to a more nuanced understanding of HR’s strategic integration in Ethiopia’s manufacturing sector, advancing both theoretical knowledge and practical methodologies in the field.

## 2. Theoretical framework

### 2.1. The conceptualization of SHRM

The concept of “strategy” forms a critical link between the foundational principles of HRM and strategic HRM, making it imperative to elucidate the fundamental tenets of SHRM. Strategic HRM establishes a coherent connection between business strategy and HRM, with a primary focus on aligning HR practices to the broader organizational strategy and its external context. The pioneers of strategic human resource management were U.S. researchers Fombrun et al. [21], who introduced the ‘Matching Model,’ and Beer et al., [22], who developed the ‘Harvard Framework.’ The Michigan Model emphasizes the need for a tight alignment between business strategy, organizational structure, and human resource management [21]. In contrast, the ‘Harvard Model’ highlights the presence of multiple stakeholders within an organization. This model adopts a softer approach to HRM, focusing on fostering employee commitment and engagement by addressing the interests of various stakeholders involved in the organization.

The integration of HRM with organizational strategy has undergone considerable transformation since the early conceptualizations of strategic HRM. In the mid-1980s, Golden and Ramanujam [23] made notable contributions to this discussion by classifying the relationship between business strategy and HRM into four categories: administrative linkage, one-way linkage, two-way linkage, and integrative linkage. This classification was further supported by Buller [24], who similarly observed a limited occurrence of integrative relationships in practice, indicating that only one out of ten organizations demonstrated a strong connection between HRM and business strategy. These empirical studies reveal a significant gap between theoretical constructs and their practical implementation within the field of HRM, emphasizing the challenges associated with effectively aligning HRM practices with broader organizational objectives.

The conceptualization of HRM’s strategic role continued to evolve with the work of Baird and Meshoulam [25], who proposed a comprehensive model for developing HRM strategies encompassing both external and internal fit. They delineated five developmental stages—initiation, functional growth, controlled growth, functional integration, and strategic integration—demonstrating that organizational effectiveness is contingent upon the alignment of HRM with the organization’s developmental phase. The initiation stage, akin to the administrative linkage described by Golden and Ramanujam, characterizes a phase where HR activities are predominantly clerical. In contrast, the strategic integration stage signifies a holistic alignment of HRM with organizational objectives across functional areas.

### 2.2. The role of HR managers in decision making

Recent scholarly work has increasingly underscored the strategic role of HRM in organizations and reinforces that HR professionals must move beyond traditional administrative roles to become strategic partners [1,26,27]. Recent empirical literature [2,7,28–32] consistently affirms that HR managers’ strategic orientation and active involvement in strategy development are crucial for organizational success. By aligning HR practices with business objectives, managing talent effectively, and driving change initiatives, HR managers play a central role in enhancing organizational performance, innovation, and long-term competitiveness. The integration of HR into strategic decision-making processes is no longer optional but essential in the dynamic, globalized business environment. However, research on strategic HRM orientations and HR roles in strategic development in developing countries specifically within Ethiopia’s manufacturing sector is notably scant, with only a few studies offering insights into this area [14,16–18], as these studies identify challenges related to capacity building, low labor quality and skill mismatches, which hinder effective strategic HR practices.

### 2.3. The theoretical framework for decision-making factors and SHRM

A significant amount of empirical research [33–38] has examined the integration of HRM with business strategy, frequently utilizing the generic business strategy models proposed by Miles and Snow [39] or the behavioral approaches suggested by Schuler and Jackson [40]. However, prominent scholars in the field argue that the traditional concept of fit is overly simplistic and naive, particularly since organizations often need to undergo significant changes in response to shifting environmental conditions [13,41–43]. As a result, any previously established fit may be disrupted. Therefore, instead of solely exploring the statistical relationship between HRM practices and the measures of generic strategies within firms, it is more relevant to assess the extent to which organizations actively pursue the integration of strategy and HRM. This perspective emphasizes the necessity for adaptability and proactive alignment in a constantly changing business environment.

Ulrich and Brockbank established four key HRM roles, which became a fundamental framework for recognizing the strategic significance of HRM within organizations. The framework stresses the need for HR professionals to evolve from traditional administrative roles into strategic partners in achieving organizational objectives [44]. It identifies four essential HRM roles: strategic partner, change agent, employee champion, and administrative expert [45]. As strategic partners, HR professionals align HR strategies with business goals, enhancing talent management, organizational culture, and employee engagement to improve overall performance [46]. This evolution reflects a broader recognition of employees as vital contributors to competitive advantage, thereby repositioning HRM as an essential driver of organizational effectiveness rather than merely an administrative function.

The theoretical framework for this study builds on the works of Brewster and Larsen [47], Budhwar and Sparrow [48], Sheehan et al. [49], and Azmi [50], who emphasized that vertical fit requires integrating HRM considerations into the formulation of organizational strategies. This underscores the need for HRM to play an active role across various organizational levels. Vertical fit, or integration, refers to the extent to which HRM is incorporated into the development of business or organizational strategies. This approach involves HR professionals participating in strategic decision-making from the earliest stages, including problem identification and the evaluation of alternative solutions. Based on these prior studies [47–50], eight key items were identified as a strong framework for evaluating HR managers’ influence on decision-making, assessed using a 5-point Likert scale, where 5 represents “strongly agree” and 1 represents “strongly disagree.”

## 3. Hypotheses

### 3.1. The convergence and divergence of HRM practices

Exploring the convergence-divergence debate within the Ethiopian manufacturing sector can provide valuable insights into whether HRM systems and practices are increasingly aligned due to economic growth, development, and the evolution of the HRM function. The convergence and divergence of HRM practices are central to the ongoing academic debate between Universalist and contingency approaches to HRM. Studies focusing on divergence emphasize the crucial relationship between environmental factors and HRM practices [51]. The foundational work of Aycan et al. [52] introduced the culture fit model (MCF), which highlights the importance of aligning HRM practices with organizational culture. According to this model, effective human resource management requires a thorough understanding of both internal organizational dynamics and external environmental factors [53]. Cultural differences are particularly significant in managing human resources in globalized settings, where adapting HR strategies to diverse socio-cultural contexts is vital for organizational success [54,55].

Early scholars supporting the divergence approach to HRM, such as, Aycan et al. [52], Hickson and Pugh [56], Hofstede [57], and Aycan et al. [58], emphasize the significant impact of national socio-economic contexts on management practices. While some HRM practices may be shared across certain contexts, others are distinctive to specific countries, sectors, or organizational divisions. Each perspective highlights particular aspects of HRM but may overlook others [59]. While there is macro-level convergence in managerial thought regarding strategic HRM concepts such as integration and development, considerable divergence remains at the micro level, where practices can differ significantly [60]. This divergence is particularly noticeable in internal work cultures shaped by managerial beliefs rooted in socio-cultural contexts, which directly influence the effectiveness of HR strategies. In developing countries like Ethiopia, these localized beliefs are essential for addressing the complexities of organizational dynamics and external socio-economic factors.

Convergence in HRM practices occurs when a unified set of political, economic, and managerial value systems standardizes management practices across organizations [61]. On the other hand, divergence emerges from substantial differences in national regulations, government policies, cultural beliefs, and other institutional factors [61]. Scholars increasingly acknowledge the impact of political systems and legal frameworks on these dynamics [59,62]. Additionally, there is growing attention to specific contextual factors—such as cultural and institutional variables—that can lead to convergence in certain cases while promoting divergence in others [63]. This understanding is particularly pertinent in developing countries like Ethiopia, where local socio-economic conditions play a significant role in shaping HRM practices. As a result, the following hypotheses are proposed:

H_O_: There is no statistically significant difference in the role of HRM in shaping decision-making across various sub-sectors, indicating uniformity in their implementation.

H_1_: There is a statistically significant difference in the role of HRM in shaping decision-making across various sub-sectors, suggesting variability in its implementation.

## 4. Materials and methods

This study employed a mixed-methods research design underpinned by pragmatic epistemology, emphasizing problem-solving and the triangulation of data sources to address the paucity of research in this field. Qualitative and quantitative datasets were analyzed independently yet concurrently, with their findings systematically integrated to yield a holistic and nuanced understanding of the phenomena under investigation. The study collected data from large and medium manufacturing companies in Addis Ababa and adjacent regions of Oromia, which together host 68% of Ethiopia’s manufacturing industries [64]. It focused on these companies, expecting them to have more strategic HRM practices than smaller firms. A total of 2,523 organizations were identified from the Ministry of Trade and Industry directory. Using Krejcie and Morgan’s [65] formula, a sample size of 334 was calculated. To address potential non-responses, Kotrlik and Higgins’ [66] oversampling method increased the final sample size to 371. Data saturation guided the determination of the appropriate number of interviewees, following Creswell’s [67] guideline of 20–30 participants for qualitative research. While 30 interviews were initially planned, a total of 24 were completed with HR directors and managers from several manufacturing sectors. Before the study began, participants received a letter from Addis Ababa University outlining the research’s purpose, voluntary nature, and privacy safeguards. Ethical standards were followed, with confidentiality assured through request letters sent to relevant parties. Participation was voluntary, with ‘verbal consent’ obtained to avoid career risks from written documentation. During data processing, participants’ names were dissociated from their responses, and pseudonyms were used for interviews to protect their identities. This ensured informed consent was given without coercion, maintaining privacy and confidentiality throughout the study.

The evaluation of strategic HRM orientation and HR roles in strategic development was based on eight items from studies by Azmi [50], Budhwar and Sparrow [48], Brewster et al. [68], and Brewster and Larsen [47]. Respondents rated their agreement on a 5-point Likert scale. The survey results were analyzed by dividing manufacturing sectors into four groups based on CSA [64] classifications: (1) Food, Beverage, and Tobacco, (2) Textile and Clothing, (3) Metal, Non-metal, and Furniture, and (4) Chemical, Pharmaceuticals, and Plastics. Data analysis was conducted using SPSS version 26, chosen for its advanced capabilities to ensure precise and reliable results. A Kruskal-Wallis Test, a non-parametric alternative to one-way ANOVA, was employed to identify significant differences in population medians among these groups utilizing SPSS version 26 [69]. This test ranks data to assess equality of medians across independent groups, making it suitable for non-parametric ordinal data analysis.

The Mann-Whitney U Test was used to evaluate differences between manufacturing sub-sectors after the Kruskal-Wallis Test. This non-parametric test, an alternative to the t-test for independent samples, compares medians instead of means [69]. It ranks scores from both groups to identify significant differences in these ranks. To control for Type I error across multiple pairwise comparisons, the p-value of 0.05 was adjusted using Bonferroni correction. This adjustment is necessary because conducting numerous tests can lead to an overall alpha level exceeding the acceptable threshold for individual tests, thus requiring stricter alpha levels for each comparison to maintain statistical integrity [69,70].

The qualitative data analysis employed thematic analysis in accordance with Creswell’s [67] five-step methodology. The process began with data preparation and organization, which included secure storage, transcription of interviews, and digitization of field notes, with all original Amharic materials translated into English for better accessibility. The second step involved an exploratory analysis of the data to facilitate coding, during which preliminary insights into key issues were identified. In the third phase, systematic coding was conducted, categorizing text and assigning specific labels that aligned with the thematic framework. The fourth step involved synthesizing these codes into overarching themes that encapsulated the core findings of the study. Finally, the fifth phase focused on interpreting these themes, allowing researchers to derive meaningful conclusions and insights, thereby enhancing the study’s validity and reliability. This rigorous approach ensures that the analysis not only captures the complexity of the data but also contributes to a deeper understanding of the research questions at hand.

## 5. Results

Of 371 questionnaires sent to HR directors and managers of manufacturers, 316 responses were received, with 282 usable; 13 were excluded due to missing data, and 21 organizations were eliminated because their CEOs managed both HR and strategic decision-making, making integration inferences unreliable [71]. Table 1 illustrates the demographic characteristics of the respondents. The data shows a clear male predominance (78.7%) in managerial (HR) positions, with females making up 21.3% of these roles in the manufacturing sector. In terms of age, 25.9% of respondents are under 30 years old, while nearly half (49.6%) are between 31 and 40 years old. Overall, 75.5% of respondents are under 40, with the majority being younger professionals. Regarding educational background, most HR professionals hold a college degree, and a notable proportion (4.6%) have completed postgraduate studies. Specifically, 83.63% of respondents possess a bachelor’s degree, and 12.10% have a certificate, meaning 87.9% of respondents in the manufacturing sector have at least a first degree or higher. As for job roles, 80.5% are HR managers, while the remaining respondents work in administrative and financial positions, also in HR-related roles.

**Table 1:** Respondents’ Demographic Characteristics.

Characteristics		Frequency	Percent
Gender	Female	60	21.3
	Male	222	78.7
	Total	282	100
Age	≤ 30 Years	73	25.9
	31–40	140	49.6
	41–50	58	20.6
	>50 Years	11	3.9
	Total	282	100
Educational levels	Certificate	34	12.1
	First Degree	235	83.3
	Second Degree	13	4.6
	Total	282	100
Positions	HR Manager	227	80.5
	Admin. and Finance	55	19.5
	Total	282	100

### 5.1. Levels of strategic HRM orientation

The foundational premise of SHRM posits that human resources should be integrated with overarching business strategies, rather than being treated as a distinct set of priorities. In alignment with this premise, the data pertaining to the levels of strategic HRM orientation and the roles of HR in strategic development is systematically presented in Table 2. The measurement framework comprises eight items that were derived from a review of relevant literature. This data was generated through crosstab analysis, complemented by the application of the Kruskal-Wallis test, a non-parametric statistical method employed to assess differences between groups. This approach not only facilitates a nuanced understanding of the interrelation between HR practices and strategic business objectives but also provides empirical evidence regarding the variability of these practices across different manufacturing contexts.

**Table 2:** Results.

Item	Sector	Strongly Disagree		Disagree		Undecided		Agree		Strongly Agree		Total		Kruskal-Wallis Test	
		N	%	N	%	N	%	N	%	N	%	N	%	Mean Rank	KW Values
**1.Human resource considered as a vital asset**	Food	2	2.9%	10	14.5%	11	15.9%	27	39.1%	19	27.5%	69	100.0%	143.25	H = 2.83 df = 3 P = 0.41
	Textile	1	1.3%	18	22.8%	1	1.3%	40	50.6%	19	241%	79	100.0%	143.18	
	Metal	8	10.4%	16	20.8%	6	7.8%	27	35.1%	20	26.0%	77	100.0%	130.15	
	chemical	3	5.3%	12	21.1%	3	5.3%	16	28.1%	23	40.4%	57	100.0%	152.38	
**Total**		14	5.0%	56	19.9%	21	7.4%	110	39.0%	81	28.7%	282	100.0%		
**2. There is a conscious effort to align business strategy with HR issues**	Food	2	2.9%	5	7.2%	8	11.6%	33	47.8%	21	30.4%	69	100.0%	166.96	H = 12.90 df = 3 P = 0.005
	Textile	8	10.1%	22	27.8%	10	12.7%	22	27.8%	17	21.5%	79	100.0%	124.82	
	Metal	10	13.0%	14	18.2%	7	9.1%	32	41.6%	14	18.2%	77	100.0%	130.73	
	chemical	3	5.3%	12	21.1%	1	1.8%	28	49.1%	13	22.8%	57	100.0%	148.34	
**Total**		23	8.2%	53	18.8%	26	9.2%	115	40.8%	65	23.0%	282	100.0%		
**3.HR inputs considered important and utilized to align with business strategy**	Food	4	5.8%	12	17.4%	4	5.8%	26	37.7%	23	33.3%	69	100.0%	146.43	H = 4.75 df = 3 P = 0.19
	Textile	2	2.5%	13	16.5%	6	7.6%	36	45.6%	22	27.8%	79	100.0%	145.10	
	Metal	5	6.5%	14	18.2%	6	7.8%	40	51.9%	12	15.6%	77	100.0%	125.64	
	chemical	4	7.0%	10	17.5%	3	5.3%	17	29.8%	23	40.0%	57	100.0%	151.97	
**Total**		15	5.3%	49	17.4%	19	6.7%	119	42.2%	80	28.4%	282	100.0%		
**4. Top management takes interest in HR issues**	Food	3	4.3%	19	27.5%	2	2.9%	24	34.8%	21	30.4%	69	100.0%	132.39	H = 1.67 df = 3 P = 0.64
	Textile	3	3.8%	17	21.5%	0	0.0%	29	36.7%	30	38.0%	79	100.0%	147.86	
	Metal	3	3.9%	10	13.0%	3	3.9%	38	49.4%	23	29.9%	77	100.0%	144.66	
	chemical	7	12.3%	9	15.8%	0	0.0%	21	36.8%	20	35.1%	57	100.0%	139.44	
**Total**		16	5.7%	55	19.5%	5	1.8%	112	39.7%	94	33.3%	282	100.0%		
**5. HR manager involvement in strategic decision makings**	Food	8	11.6%	13	18.8%	8	11.6%	29	42.0%	11	15.9%	69	100.0%	146.33	H = 12.1 df = 3 P = 0.007
	Textile	3	3.8%	23	29.1%	1	1.3%	31	39.2%	21	26.6%	79	100.0%	162.36	
	Metal	10	13.0%	26	33.8%	4	5.2%	23	29.9%	14	18.2%	77	100.0%	133.80	
	chemical	13	22.8%	17	29.8%	2	3.5%	19	33.3%	6	10.5%	57	100.0%	117.14	
**Total**		34	12.1%	79	28.0%	15	5.3%	102	36.2%	52	18.4%	282	100.0%		
**6. HR manager consulted from the outset at the development of organizational strategy**	Food	19	27.5%	11	15.9%	4	5.8%	23	33.3%	12	17.4%	69	100.0%	125.23	H = 25.2 df = 3 P = 0.000
	Textile	1	1.3%	16	20.3%	4	5.1%	27	34.2%	31	39.2%	79	100.0%	178.53	
	Metal	4	5.2%	38	49.4%	11	14.3%	12	15.6%	12	15.6%	77	100.0%	122.05	
	chemical	9	15.8%	19	33.3%	1	1.8%	12	21.1%	16	28.1%	57	100.0%	163.16	
**Total**		33	11.7%	84	29.8%	20	7.1%	74	26.2%	71	25.2	282	100.0%		
**7. Existence of a written HR strategy**	Food	10	14.5%	28	40.6%	3	4.3%	22	31.9%	6	8.7%	69	100.0%	147.80	H = 3.16 df = 3 P = 0.36
	Textile	11	13.9%	39	45.6%	1	1.3%	24	30.4%	7	8.9%	79	100.0%	145.13	
	Metal	23	29.9%	23	29.9%	5	6.5%	21	27.3%	5	6.5%	77	100.0%	128.15	
	chemical	11	19.3%	21	36.8%	1	1.8%	16	28.1%	8	14.0%	57	100.0%	146.88	
**Total**		55	19.5%	108	38.3%	10	3.5%	83	29.4%	26	9.2%	282	100.0%		
**8. HR strategy is translated into a clear set of workable programs**	Food	21	30.4%	21	30.4%	0	0.0%	17	24.6%	10	14.5%	69	100.0%	149.59	H = 1.80 df = 3 P = 0.61
	Textile	24	30.4%	34	43.0%	4	5.1%	12	15.2%	5	6.3%	79	100.0%	132.68	
	Metal	21	27.3%	30	39.0%	3	3.9%	20	26.0%	3	3.9%	77	100.0%	141.66	
	chemical	17	29.8%	19	33.3%	3	5.3%	13	22.8%	5	8.8%	57	100.0%	143.71	
**Total**		83	29.4%	104	36.9%	10	3.5%	62	22.0%	23	8.2%	282	100.0%		

Many companies today are increasingly recognizing the importance of their workforce in achieving organizational goals and maintaining a competitive edge. HRM is critical to organizational success because human capital possesses qualities that make it valuable for sustainable competitive advantage. In line with this, respondents were asked to rate their level of agreement on whether “human resources are considered a vital asset” in the manufacturing sector. The results indicate that 39% of respondents agreed and 28.7% strongly agreed with the statement, meaning that a majority (67.7%) believe human resources are indeed a vital asset in their organizations (see Table 2). However, 19.9% disagreed and 5.0% strongly disagreed, suggesting that a minority does not share this perspective. While the general consensus supports the view that human capital is key to organizational success, the minority’s disagreement points to possible gaps in HRM practices or organizational culture. These differences may reflect challenges in aligning HR strategies with business goals or in communicating the strategic importance of HR to all organizational levels. As a result, while most organizations value their human resources, there is room for improvement in fostering a more universally shared understanding of HR’s role in driving competitive advantage within the manufacturing sector.

The Kruskal-Wallis test was conducted to determine if there were any significant differences between four groups of manufacturing sectors in terms of how much they considered “human resources as a vital asset.” The test yielded a P-value of 0.41 (H = 2.83, df = 3), indicating no statistically significant difference in the level of agreement among the groups. This suggests that the sectors generally hold similar views regarding the importance of human resources. However, the mean rank values provide some insight into sector-specific tendencies. As shown in Table 2 respondents from the chemical and pharmaceutical sectors had the highest mean rank (152.38), indicating a stronger inclination to agree that human resources are a vital asset. In contrast, respondents from the metal, non-metal, and furniture sectors had the lowest mean rank (130.15), suggesting less agreement on this point. The other two sectors, food, beverages, and tobacco (mean rank 143.25), and textile and clothing (mean rank 143.18), were more closely aligned in their perspectives. While no significant differences were found statistically, these mean ranks reflect slight variations in how different sectors value human resources.

The interview findings shed light on the factors that underscore the importance of human resources as a crucial asset within the manufacturing sector. Respondents noted that the current competitive landscape, combined with government initiatives aimed at promoting equal employment opportunities, has increased the necessity for companies to emphasize human capital as a central element of success. This aligns with modern human resource management perspectives, which contend that organizations that prioritize and effectively manage their employees are better positioned for sustained success in competitive environments. Furthermore, the government’s focus on fair employment practices serves as a catalyst, prompting organizations to adopt more inclusive and strategic approaches to workforce management. These external pressures, along with the need to maintain competitiveness, are driving organizations to recognize the significance of their workforce in a more organized and strategic manner.

The consistency between the interview findings and the survey data reveals a sector-wide acknowledgment of the importance of human resources. However, it also suggests that the level of HR maturity may vary between sectors. While some industries, like food and beverages, seem to have well-established HR practices, others may still be developing these systems. This implies an opportunity for sectors with lower HR engagement, such as the metal and non-metal categories, to learn from best practices and further integrate HR as a strategic partner in their operations. In conclusion, the interviews confirm that human resources are increasingly seen as vital, and that the strategic management of employees is becoming a competitive necessity across the manufacturing sector.

Respondents from the manufacturing sector were asked to express their level of agreement regarding the extent to which there is a deliberate effort to align business strategy with HR issues. In this context, 115 respondents (40.8%) agreed, and 65 respondents (23.0%) strongly agreed with the statement about this alignment, collectively representing the majority, or 180 respondents (63.8%) (Table 2). This positive response indicates recognition of the importance of integrating HR strategies with business objectives, which can enhance organizational effectiveness. Conversely, 76 respondents (27%) disagreed or strongly disagreed with the statement, highlighting a notable minority that perceives a disconnect between HR practices and business strategy. Additionally, the remaining 9.2% of respondents were undecided, suggesting uncertainty or lack of engagement with this issue. Collectively, these findings suggest that while many in the manufacturing sector acknowledge efforts to align HR with business strategy, there remains a significant portion of the workforce that identifies potential gaps, underscoring the need for improved communication and collaboration in this area.

The Kruskal-Wallis test yielded a p-value of 0.005 (H = 12.9, df = 3), indicating that the levels of agreement among the four groups of manufacturing sectors are significantly different from one another. Consequently, the null hypothesis, which posits that the population medians for the four groups are equal, is rejected. To further investigate these differences, the Mann-Whitney test was employed for appropriate pair-wise comparisons among the manufacturing sectors (see Table 3). As detailed in the methodology section of this dissertation, the Mann-Whitney test is a non-parametric method that is analogous to conducting a standard parametric two-sample t-test on ranked data from combined samples [69].

**Table 3:** Comparative Pair-wise Analysis of Effort to Align Business Strategy with HR issues.

	Food categories *versus* Textile categories	Food categories *Versus* Metal categories	Food categories *versus* Chemical categories	Textile categories *versus* Metal categories	Textile categories *versus* Chemical categories	Metal categories *versus* Chemical categories
Mann-Whitney U	1904.500	1977.000	1710.500	2913.000	1883.500	1916.500
Sig. (2-tailed)	0.001	0.005	0.177	0.638	0.091	0.186

The results presented in Table 3 indicate that there are statistically significant differences in responses between specific groups in the manufacturing sector based on the Mann-Whitney test. Notably, the responses from the food, tobacco, and beverage sector (Group One) were significantly different from those in the textile and clothing sector (Group Two) with a P-value of 0.001, indicating a strong statistical significance. Similarly, the food, tobacco, and beverage sector also showed significant differences in responses compared to the metal, non-metal, and furniture sector, with a P-value of 0.005 level of significance. This suggests their response regarding effort to align business strategy with HR issues differ notably between these specific sectors. However, the Mann-Whitney test results for the other four pairwise comparisons among the subsectors revealed P-values of 0.177, 0.638, 0.091, and 0.186, indicating that there were no significant differences among those groups.

The semi-structured interviews revealed mixed results concerning the alignment of organizational strategies with human resource issues. While some informants acknowledged that human resource managers play a crucial role in this alignment, they emphasized that such efforts cannot succeed without the active support and commitment of chief executive officers (CEOs). This highlights a critical insight: the alignment of business strategy with human resource issues is a collaborative process that requires the involvement of top leadership. Informants pointed out the growing challenge of securing the willingness of general managers to engage in this alignment, suggesting that there may be organizational barriers or a lack of awareness regarding the importance of such integration.

This analysis underscores that effective human resource management encompasses not only the functions performed by HR professionals but also the necessity of a supportive organizational culture and active leadership commitment. The diverse responses indicate that, although HR managers are making strides in aligning strategies, their efforts may be impeded by a lack of engagement from senior management. This situation suggests a potential disconnect between HR initiatives and overarching business objectives, which could adversely impact organizational performance. Ultimately, the findings emphasize that fostering an environment conducive to alignment requires a collaborative effort between HR and executive leadership, highlighting the importance of cultivating a culture that acknowledges the strategic role of human resources in facilitating organizational success.

A question was raised to measure the extent to which human resource inputs are seen as an essential component of business or organizational strategy. The findings showed that 42.2% of respondents agreed, and 28.4% strongly agreed with the statement, making a combined 70.64% of respondents who believe that HR inputs are integral to their organizational strategies (Table 2). On the other hand, 17.46% of respondents disagreed, 5.3% strongly disagreed, and 6.7% were undecided. These results highlight a clear majority of manufacturing sector respondents who recognize the pivotal role human resources play in shaping business strategy. This is an encouraging trend, as it signals an increasing appreciation for the strategic value of HR, a critical factor in the development and implementation of SHRM. The widespread agreement supports the idea that many organizations in this sector are moving toward more integrated HR practices that contribute directly to organizational success. Furthermore, the Kruskal-Wallis test, which produced a P-value of 0.19 (H = 4.75, df = 3), indicated no significant difference in the levels of agreement across the four groups of manufacturing sectors.

The key informant interviews support the survey findings, indicating that human resource inputs are increasingly valued in the organizational strategy development process across the manufacturing sector. Most interviewees emphasized the importance of utilizing HR data, such as employee numbers, qualifications, professions, and experience, in formulating strategies. This reflects a shift toward a more data-driven and strategic approach to human resource management, where workforce capabilities are aligned with broader business objectives. The integration of detailed employee information into strategic planning demonstrates an awareness of the critical role human resources play in organizational success and competitive advantage, highlighting the growing influence of HR in driving long-term business outcomes.

Another question was asked to assess top management’s interest in HR issues within the manufacturing sector. The findings reveal that 73% of respondents believe top management attaches significant importance to HR, indicating a positive shift toward recognizing HR’s strategic role in achieving organizational success (Table 2). This widespread agreement implies that executives are increasingly integrating HR with key business strategies to improve operational efficiency and gain a competitive edge. However, 25.2% of respondents expressed disagreement, suggesting some inconsistencies in how different organizations prioritize HR. The Kruskal-Wallis test (P-value of 0.64) indicates no significant differences in perceptions among the four manufacturing sectors, pointing to a general consistency of this viewpoint across industries. Although responses are relatively uniform, the presence of dissenting opinions reveals opportunities for leadership to enhance their engagement with HR, ensuring a more consistent strategic alignment throughout the sector.

The results from interviews with key informants align with earlier findings, highlighting the undeniable interest that top management has in HR issues. Informants underscored that human resources are fundamental assets for organizations, and their associated costs significantly impact budgetary considerations. This insight reflects a shared perspective among HR managers across various sectors regarding the necessity of top management involvement as a crucial factor for effectively aligning HR strategies with organizational objectives. Specifically, informants pointed out that during the long-term strategy formulation process, managers prioritize the significance of human resources in extracting sensitive information that can greatly influence organizational outcomes. This indicates that respondents recognize an increasing interest from top management in HRM matters, suggesting a shift towards valuing HR as a strategic partner in organizational success.

### 5.2. HR managers roles in shaping decision making

The extent to which the human resource function participates in both organizational and HR strategy development is influenced by various factors, notably the presence of the senior human resource manager on the board of directors. The involvement of HR managers in key organizational decisions is vital, as it can have a significant impact on overall policies. To evaluate this involvement, respondents were asked to express their level of agreement regarding the participation of HR managers in strategic decision-making. The result in Table 2, reveals that 36.2% and 18.4% of respondents agreed and strongly agreed, respectively, indicating that a total of 54.6% support the inclusion of HR managers in strategic decisions. In contrast, 40.1% of respondents disagreed (comprising 28.0% who disagreed and 12.1% who strongly disagreed), while 5.3% were undecided. This finding indicate that, despite recognizing human resources as essential assets within organizations, the complete integration of strategic human resource management—specifically regarding HR managers’ involvement in decision-making processes—has not yet been fully realized. This gap highlights the need for organizations to strengthen the role of HR in strategic discussions to effectively capitalize on their contributions to organizational success.

According to the CRANET International Executive Reports from 2021–2022, 71% of organizations globally have a person responsible for HRM serving on the board [72]. CRANET, established in 1989, is a global network of business schools that conducts a survey every five years on human resource management policies and practices. The 2017 CRANET survey found that Sweden and Spain had the highest involvement of HR managers in organizational decision-making, with 89.0% and 84.7% involvement, respectively [73]. In non-EU countries, HR manager representation in board decisions averaged between 60% and 70%, while in non-European nations, it ranged from 52.5% in South Africa to 75.2% in China. However, 54.6% of respondents in the Ethiopian manufacturing sector agreed or strongly agreed that HR managers participate in strategic decision-making.

A Kruskal-Wallis test revealed notable differences in HR managers’ involvement in decision-making across manufacturing sectors (p = 0.007, H = 12.1, df = 3), leading to the rejection of the null hypothesis of equal medians.

Pairwise comparisons conducted using the Mann-Whitney test, as shown in Table 4, revealed significant differences between the textile and chemical sectors (p = 0.001) as well as between the food and chemical sectors (p = 0.036). The other comparisons were not significant, with p-values ranging from 0.188 to 0.311. The textile and clothing sector had the highest mean rank (162.36), while the chemical and pharmaceutical sectors had the lowest (117.14).

**Table 4:** Comparative Pair-wise Analysis of HR Representation.

	Food categories *versus* Textile categories	Food categories *Versus* Metal categories	Food categories *versus* Chemical categories	Textile categories *versus* Metal categories	Textile categories *versus* Chemical categories	Metal categories *versus* Chemical categories
Mann-Whitney U	2398.5	2406.5	1556	2434.5	1537.5	1930.5
Sig. (2-tailed)	0.188	0.311	0.036	0.24	0.001	0.219

Human resource managers are essential to the development of organizational strategies, and their engagement can greatly affect HR-related decision-making. Their participation in strategy formulation increases the likelihood that HR issues will be adequately addressed and integrated into the overall organizational framework. Nevertheless, insights from key informant interviews reveal that, despite the presence of HR executives in senior management, many believe that this representation does not guarantee a strategic impact. This belief is often linked to the lack of strong connections between HR managers and CEOs. When HR managers are not included in decision-making processes, their strategies tend to be reactive, adjusting to the decisions of top management instead of contributing to the strategy’s formulation. Consequently, it is vital to regard HR managers and their departments as strategic assets rather than merely administrative functions centered on compliance and cost control. To foster this perspective, senior leaders must recognize the strategic role of HR and involve it in their strategic planning processes.

Another question sought to measure the extent to which HR managers are involved from the beginning in the formulation of organizational strategy. The results in Table 2 reveal that 26.2% (74 respondents) and 25.2% (71 respondents) agreed and strongly agreed with the statement that “human resource managers are consulted from the outset in the development of organizational strategy.” This suggests that approximately half of the respondents (51.4%) believe HR managers are included early in the strategic planning process. Conversely, 29.8% (84 respondents) and 11.7% (33 respondents) disagreed and strongly disagreed, indicating that a total of 41.5% feel HR managers are not consulted from the start. Additionally, 7.1% (20 respondents) remained undecided on the matter. While just over half of the respondents see HR managers as integral to the early stages of strategy development, a significant minority believes they are excluded from these critical discussions. This divide may reflect underlying issues in organizational culture and communication, highlighting a potential gap in recognizing the strategic value of HR within manufacturing sectors.

A Kruskal-Wallis test was conducted to evaluate differences in the involvement of HR managers across four manufacturing sectors, yielding a significant result (p = 0.000, H = 25.2, df = 3). Mann-Whitney tests in Table 5, revealed significant disparities in HR consultation between the food, beverage, and tobacco sectors and the textile and clothing sector (p = 0.000), as well as between the textile/clothing and metal/non-metal/furniture sectors (p = 0.000) and the chemical/pharmaceutical sectors (p = 0.004). These findings highlight sectoral variations in recognizing HR’s strategic role, underscoring the need for a more consistent approach to enhance organizational effectiveness. This variation underscores the differing recognition of HR’s strategic contributions across sectors, suggesting a need for a more uniform approach to involving HR managers in strategic discussions to enhance overall organizational effectiveness and alignment.

**Table 5:** Comparative Pair-wise Analysis of HR consultation from the outset.

	Food categories *versus* Textile categories	Food categories *Versus* Metal Categories	Food categories *versus* Chemical categories	Textile categories *versus* Metal categories	Textile categories *versus* Chemical categories	Metal categories *versus* Chemical categories
Mann-Whitney U	1740	2623	1796	1729	1624.5	2042.5
Sig. (2-tailed)	0.000	0.894	0.392	0.000	0.004	0.472

The results from key informant interviews contrast sharply with previous findings, revealing that most interviewed managers view the human resource function as having a minimal role in the formulation of organizational strategy. Instead, they perceive HR’s primary function as supporting the execution of business strategies that are established by top management. This perspective highlights a critical issue: while early involvement of HR managers in the strategic decision-making process is essential for enabling HR professionals to influence board decisions, the current lack of such involvement relegates HR to merely executing decisions made by executives. This disconnect underscores a significant opportunity for organizations to rethink the integration of HR in strategy development.

The establishment of a written human resource strategy that aligns with organizational strategic goals is crucial for the effective planning of HRM activities and practices. HR strategies are designed to clarify an organization’s intentions regarding its human resource management policies and practices, both in the present and for the future. To evaluate the presence of such strategies, respondents were asked to indicate their level of agreement or disagreement regarding the existence of a formal HR strategy. The findings revealed that a significant majority, specifically 57.8%, disagreed (38.3%) or strongly disagreed (19.5%) with the assertion that a written human resource strategy is in place (Table 2). This suggests that over half of the respondents in the manufacturing sector believe their HR strategies lack official documentation. Conversely, only 29.4% (83 respondents) agreed, and 9.2% (26 respondents) strongly agreed that such a written strategy exists, totaling 38.6%. Additionally, 10 respondents, or 3.5%, remained undecided. This points to a notable deficiency in documented human resource strategies within the manufacturing sectors, despite the growing recognition of such documentation as an essential element of overall organizational strategy in today’s environment. To assess whether there are significant differences among the four groups of manufacturing sectors, the Kruskal-Wallis test was applied. The findings revealed no significant differences, with a p-value of 0.36 (H = 3.165, df = 3).

The semi-structured interviews conducted with HR managers highlighted a significant absence of written HR strategies within their departments. While nearly half of the HR managers participated in senior-level committee meetings, they reported challenges in translating HR strategies into actionable programs, resulting in limited consistency between HR policies and the overall organizational strategy. This suggests that HR managers are primarily focused on the daily operations of human resource functions rather than on creating formal documentation that aligns the organization’s strategic goals with its HR strategy. Despite lacking formal written strategies, HR managers indicated that they maintain both formal and informal communications with top executives to integrate HR issues into the organizational strategy and connect various HR policy areas. It is clear that organizations with documented HR strategies are better positioned to develop effective HR policies and practices that are aligned with their business strategies.

Among the eight scales assessing the integration of HRM with organizational strategy, the translation of human resource management strategy into a clear set of actionable programs was identified as the most critical factor. To gauge this, respondents rated their level of agreement regarding how effectively their organizations translate HR strategy into practical programs. The questionnaire results (Table 2) revealed that a notable 66.3% of participants—comprising 36.9% who disagreed and 29.4% who strongly disagreed—reported that their organizations do not successfully transform HR strategy into defined, workable programs. This finding aligns with earlier results indicating a limited presence of formal HR strategies in the manufacturing sectors of Addis Ababa and adjacent region of Oromia. In contrast, descriptive statistics showed that only 30.2% of respondents (22.0% agreed and 8.2% strongly agreed) believe their organizations effectively translate HR strategy into actionable programs. Furthermore, a Kruskal-Wallis test was conducted to explore potential significant differences among the four groups of manufacturing sectors, revealing no significant differences, with a p-value of 0.61 (H = 1.80, df = 3) at the 0.05 level of significance.

The results from semi-structured interviews with HR managers highlight considerable challenges in converting HR strategies into actionable initiatives that align with organizational strategy. This observation is supported by survey data, which revealed that 66.3% of participants reported difficulties in translating HR strategies into clear, practical programs. The misalignment between HR policies and organizational objectives indicates that HR functions often adopt a reactive stance, prioritizing daily operations over strategic initiatives. This gap reveals a critical shortfall in the capacity of HR departments to shape organizational direction, emphasizing the need for a more comprehensive and documented HR strategy. Furthermore, the limited involvement of HR managers in strategic decision-making, as noted in both interviews and surveys, underscores the importance of top management recognizing HR’s strategic significance. By encouraging the early and meaningful engagement of HR professionals in strategy formulation, organizations can improve HR effectiveness and ensure alignment with broader business goals, ultimately enhancing overall organizational performance.

### 5.3. Summary of key insights from identified themes

The analysis of qualitative data from interviews revealed eight key themes that highlight the multifaceted role of HR managers into strategic decision-making. These themes provide a nuanced understanding of the current state of HR practices within the manufacturing sector, emphasizing the persistent challenges in aligning HR functions with broader organizational strategies. While HR is increasingly acknowledged as a vital organizational asset, its integration into strategic planning remains constrained by systemic barriers. Key issues include the limited early involvement of HR professionals in decision-making processes, the absence of formalized HR strategies, and a predominantly reactive approach to aligning HR with business objectives. Additionally, the findings highlight the pivotal role of leadership commitment and organizational culture in defining HR’s strategic influence. Without robust support from senior leadership, HR’s capacity to drive organizational outcomes is significantly diminished. Nonetheless, there is a notable shift toward data-driven HR management, reflecting a growing awareness of the need to align workforce capabilities with strategic priorities. These insights point to the urgency for organizations to address structural and cultural impediments to fully harness the strategic potential of their human resources. Table 6 presents a summary of the key themes identified from the qualitative data, along with their corresponding descriptions.

**Table 6:** Summary of key themes.

	Themes	Description
1	Human Resources as a Strategic Asset	HR is viewed as a critical asset within the manufacturing sector, with companies increasingly recognizing its strategic value for organizational success
2	Sectoral differences in HR Maturity	Varying levels of HR maturity are noted across different sectors
3	Challenge in alignment between HR and Organizational Strategy	The alignment of HR strategies with business objectives is seen as essential but often challenging
4	Leadership Commitment and Organizational Culture	Successful HR strategy alignment is dependent on strong leadership commitment and an organizational culture that recognizes HR’s strategic role
5	Data-Driven HR Management	There is an increasing reliance on data-driven HR management, with HR data (e.g., employee numbers, qualifications, experience) being integrated into strategic decision-making
6	Need for Early Involvement of HR in Strategy Formulation	HR managers are not included early in decision-making processes, limiting their ability to influence organizational strategy.
7	Absence of Written HR Strategies	A significant number of HR departments lack written HR strategies, which impacts the ability to align HR policies with the organization’s overarching strategic goals
8	Challenges in Translating HR Strategies into Actionable Initiatives	The challenges reflect a broader issue of HR departments adopting a reactive role, focusing more on day-to-day operations than on strategic initiatives

## 6. Discussion

Given that this study developed a framework grounded in existing literature to evaluate the role of HR managers in shaping decision-making, there is a notable scarcity of direct comparative studies in the current research landscape. Nonetheless, the study draws upon pertinent empirical evidence from related fields to enrich and contextualize its findings, thereby contributing to a broader understanding of HR’s strategic involvement in decision-making processes. This approach allows the study to bridge the gap in the literature, offering valuable insights despite the limited direct comparisons available in prior research.

The findings of this study both corroborate and expand upon the issues identified in the existing research gap concerning strategic HRM orientations and the role of HR in decision-making within Ethiopia’s manufacturing sector. Prior investigations have delineated challenges such as low labor productivity, inadequate educational attainment, and the persistent reliance on traditional HR practices in the manufacturing sector [14,17,19,20]. These operational deficiencies are further substantiated in the present study, which identifies the absence of formal HR strategies across various sectors as a significant impediment. However, this research advances beyond these preliminary observations by elucidating sectoral disparities in HR’s strategic involvement. For instance, labor-intensive sectors, such as textiles, exhibit higher levels of HR engagement compared to capital-intensive industries, such as chemicals, where HR’s involvement remains minimal. This finding suggests that the strategic integration of HR is highly contingent upon the operational focus and perceived value of human capital within each sector [3].

Additionally, the findings highlight a widespread deficiency in formal HR strategies across various sectors, which significantly limits HR’s ability to improve organizational effectiveness. This aligns with existing literature that suggests that in developing nations such as Ethiopia, limited resources and a lack of awareness often obstruct the formalization of HR processes, leading to a reactive approach to HR management instead of a proactive one [74,75]. The reactive approach to HR management is a significant barrier to improving organizational performance, as it focuses on addressing short-term challenges rather than aligning HR practices with long-term strategic goals. This deficiency is particularly prevalent in sectors where HR is viewed primarily as an administrative function rather than as a strategic partner, which further compounds the challenges of aligning HR with overall business strategies.

The lack of formal HR strategies, as noted by Storey, Ulrich, and Wright [3], points to a broader issue concerning the inconsistent institutionalization of HR practices, which is further exacerbated by inadequate training and development programs [76]. The absence of structured, formal HR strategies in organizations hampers HR’s potential to contribute to the strategic decision-making process, as HR professionals are not equipped with the necessary tools and frameworks to make a significant impact. Without sufficient training and development, HR professionals are unable to transition from a reactive, operational role to a more proactive, strategic role that can help drive organizational effectiveness. Together, these results enhance our understanding of HR’s strategic role, indicating that both sectoral differences and the absence of formal HR strategies serve as significant obstacles to effectively leveraging HR’s potential in Ethiopia’s manufacturing sector.

## 7. Conclusion

### 7.1. Managerial and practical implications

These findings carry important implications for both practice and policy in the manufacturing sector. Organizations must recognize that while there is an emerging acknowledgment of the strategic role of HR, the absence of formalized HR strategies undermines their potential impact. Based on the findings of this research, several recommendations can be made to enhance the strategic role of HR in the manufacturing sector. First, it is essential for organizations to prioritize the development and formalization of HR strategies that are closely aligned with organizational objectives. This alignment will not only enhance the consistency and effectiveness of HR practices but also ensure that human resources contribute meaningfully to the strategic direction of the company. Additionally, HR managers should be integrated into the decision-making processes from the outset of strategic discussions. This will facilitate a more holistic approach to organizational strategy, ensuring that HR insights are factored into key decisions. Moreover, the existing disparity in HR representation across different sectors necessitates tailored interventions to increase the involvement of HR professionals in strategic decision-making, particularly in areas where their influence is currently limited. Organizations must continue to recognize HR as a strategic partner, elevating its role beyond administrative functions. In this regard, capacity-building initiatives aimed at equipping HR professionals with the necessary skills and knowledge to contribute strategically should be a priority. Finally, it is crucial to bridge the gap between strategic intent and the implementation of HR practices. Ensuring that HR practices are aligned with strategic goals will be fundamental to achieving sustained competitive advantage, allowing organizations to leverage their human resources as a key driver of long-term success in the manufacturing sector.

### 7.2. Limitations and further research directions

A limitation of this study is its sole focus on the manufacturing sector. Future research should examine differences across various industry subsectors to assess the role of HRM in shaping decision-making and its alignment with organizational performance. Additionally, to draw causal conclusions, it is recommended that these findings be replicated using a longitudinal design across different sectors, as the cross-sectional nature of this study restricts causal conclusions. Addressing these factors will contribute to the improvement of HR practices and enhance organizational outcomes within the manufacturing sector. Moreover, future research should focus on identifying best practices for HR integration in both labor- and capital-intensive industries, as well as examining how HR practices can be more systematically institutionalized to ensure that HR plays a proactive role in shaping organizational strategy.

### 7.3. Conclusion

In conclusion, this research underscores the critical recognition of human resources as a valuable asset within the manufacturing sectors, while also revealing significant inconsistencies in the involvement and contributions of HR managers in the strategic development process. To harness the full value of human resources, companies should prioritize the development and documentation of clear HR strategies that align with organizational goals. This formalization will not only enhance the consistency and effectiveness of HR practices but also foster a more integrated approach to decision-making, where HR managers can contribute meaningfully from the outset of strategic discussions. Additionally, the disparity in HR representation across sectors calls for tailored interventions to enhance the involvement of HR professionals in strategic decision-making processes, particularly in areas where their presence is currently limited. By actively engaging HR managers in strategic dialogues, organizations can ensure that HR insights are considered, leading to more comprehensive and effective strategies. Ultimately, bridging the gap between strategic intent and actionable HR practices will be essential for achieving sustained competitive advantage in the manufacturing sector.

## Supporting information

S1 Appendix Methods and sampling. (DOCX)

S2 Appendix Variables and Measures. (DOCX)

S3 Appendix Dataset utilized for Analysis. (DOCX)
