# Supplementary material for: The strategic role of human resource managers in shaping decision-making in Ethiopia
Source: PLoS One. 2025 Jul 8;20(7):e0327296. doi: 10.1371/journal.pone.0327296 (PMC12237031; doi:10.1371/journal.pone.0327296)
Supplement: S1 Appendix — (DOCX) [file pone.0327296.s001.docx]

**S1 Appendix Methods and Sampling**

The sample size was calculated using the finite population formula developed by Krejcie & Morgan (1970, p. 607).

$$S=\frac{X^{2}NP(1-P)}{D^{2}\left( N-1 \right)+X^{2}P(1-p)}$$

Where: S represents the required sample size; X² denotes the chi-square value for 1 degree of freedom at a 0.05 confidence level (3.841); N is the population size; P refers to the population proportion, commonly assumed to be 0.50 to maximize the sample size; and d is the margin of error or desired accuracy, expressed as a proportion (0.05 in this case).

$\frac{3.841 x 2523 x (0.5 x 0.5)}{{0.05}^{2}\left( 2523-1 \right)+ 3.841 (0.5 x 0.5)}=\frac{2422.71}{6.30+0.96025}=\frac{2422.71}{7.26}$ = **333.70**

To account for potential non-responses rate oversampling procedures proposed by Kotrlik, and Higgins (2001) was used, bringing the final sample size to 371.
