# Supplementary material for: The strategic role of human resource managers in shaping decision-making in Ethiopia
Source: PLoS One. 2025 Jul 8;20(7):e0327296. doi: 10.1371/journal.pone.0327296 (PMC12237031; doi:10.1371/journal.pone.0327296)
Supplement: S2 Appendix — (DOCX) [file pone.0327296.s002.docx]

| **S2 Appendix. Variables and measures** | | |
| --- | --- | --- |
| **Variables** | **Items** | **Sources** |
| **Integrating HRM with organizational strategy (The role of HR managers in shaping decision-making)** | Human resource considered as a vital asset | Azmi (2011)  Huselid (1995) |
|  | There is a conscious effort to align business strategy with HR issues |  |
|  | HR inputs considered important and utilized to align with business strategy |  |
|  | Top management takes interest in HR issues |  |
|  |  |  |
|  | HR manager involvement in strategic decision makings | Budhwar and Sparrow, 1997, Brewster and Hegewisch, 1994; Brewster and Larsen, 1992 |
|  | HR manager consulted from the outset at the development of organizational strategy |  |
|  | Existence of a written HR strategy |  |
|  | HR strategy is translated into a clear set of workable programs |  |
