# Supplementary material for: The strategic role of human resource managers in shaping decision-making in Ethiopia
Source: PLoS One. 2025 Jul 8;20(7):e0327296. doi: 10.1371/journal.pone.0327296 (PMC12237031; doi:10.1371/journal.pone.0327296)
Supplement: S3 Appendix — (DOCX) [file pone.0327296.s003.docx]

**S3 Dataset Utilized for Analyzing Descriptive statistics, the Kruskal-Wallis Test and Mann-Whitney Test**

| **Table 2: Results** | | | | | | | | | | | | | |
| --- | --- | --- | --- | --- | --- | --- | --- | --- | --- | --- | --- | --- | --- |
| **Item** | **Sector** | **Strongly Disagree** | | **Disagree** | | **Undecided** | | **Agree** | | **Strongly Agree** | | **Total** | |
|  |  | N | % | N | % | N | % | N | % | N | % | N | % |
| **1.Human resource considered as a vital asset** | Food | 2 | 2.9% | 10 | 14.5% | 11 | 15.9% | 27 | 39.1% | 19 | 27.5% | 69 | 100.0% |
|  | Textile | 1 | 1.3% | 18 | 22.8% | 1 | 1.3% | 40 | 50.6% | 19 | 241% | 79 | 100.0% |
|  | Metal | 8 | 10.4% | 16 | 20.8% | 6 | 7.8% | 27 | 35.1% | 20 | 26.0% | 77 | 100.0% |
|  | chemical | 3 | 5.3% | 12 | 21.1% | 3 | 5.3% | 16 | 28.1% | 23 | 40.4% | 57 | 100.0% |
| **Total** | | 14 | 5.0% | 56 | 19.9% | 21 | 7.4% | 110 | 39.0% | 81 | 28.7% | 282 | 100.0% |
| **2. There is a conscious effort to align business strategy with HR issues** | Food | 2 | 2.9% | 5 | 7.2% | 8 | 11.6% | 33 | 47.8% | 21 | 30.4% | 69 | 100.0% |
|  | Textile | 8 | 10.1% | 22 | 27.8% | 10 | 12.7% | 22 | 27.8% | 17 | 21.5% | 79 | 100.0% |
|  | Metal | 10 | 13.0% | 14 | 18.2% | 7 | 9.1% | 32 | 41.6% | 14 | 18.2% | 77 | 100.0% |
|  | chemical | 3 | 5.3% | 12 | 21.1% | 1 | 1.8% | 28 | 49.1% | 13 | 22.8% | 57 | 100.0% |
| **Total** | | 23 | 8.2% | 53 | 18.8% | 26 | 9.2% | 115 | 40.8% | 65 | 23.0% | 282 | 100.0% |
| **3.HR inputs considered important and utilized to align with business strategy** | Food | 4 | 5.8% | 12 | 17.4% | 4 | 5.8% | 26 | 37.7% | 23 | 33.3% | 69 | 100.0% |
|  | Textile | 2 | 2.5% | 13 | 16.5% | 6 | 7.6% | 36 | 45.6% | 22 | 27.8% | 79 | 100.0% |
|  | Metal | 5 | 6.5% | 14 | 18.2% | 6 | 7.8% | 40 | 51.9% | 12 | 15.6% | 77 | 100.0% |
|  | chemical | 4 | 7.0% | 10 | 17.5% | 3 | 5.3% | 17 | 29.8% | 23 | 40.0% | 57 | 100.0% |
| **Total** | | 15 | 5.3% | 49 | 17.4% | 19 | 6.7% | 119 | 42.2% | 80 | 28.4% | 282 | 100.0% |
| **4. Top management takes interest in HR issues** | Food | 3 | 4.3% | 19 | 27.5% | 2 | 2.9% | 24 | 34.8% | 21 | 30.4% | 69 | 100.0% |
|  | Textile | 3 | 3.8% | 17 | 21.5% | 0 | 0.0% | 29 | 36.7% | 30 | 38.0% | 79 | 100.0% |
|  | Metal | 3 | 3.9% | 10 | 13.0% | 3 | 3.9% | 38 | 49.4% | 23 | 29.9% | 77 | 100.0% |
|  | chemical | 7 | 12.3% | 9 | 15.8% | 0 | 0.0% | 21 | 36.8% | 20 | 35.1% | 57 | 100.0% |
| **Total** | | 16 | 5.7% | 55 | 19.5% | 5 | 1.8% | 112 | 39.7% | 94 | 33.3% | 282 | 100.0% |
| **5. HR manager involvement in strategic decision makings** | Food | 8 | 11.6% | 13 | 18.8% | 8 | 11.6% | 29 | 42.0% | 11 | 15.9% | 69 | 100.0% |
|  | Textile | 3 | 3.8% | 23 | 29.1% | 1 | 1.3% | 31 | 39.2% | 21 | 26.6% | 79 | 100.0% |
|  | Metal | 10 | 13.0% | 26 | 33.8% | 4 | 5.2% | 23 | 29.9% | 14 | 18.2% | 77 | 100.0% |
|  | chemical | 13 | 22.8% | 17 | 29.8% | 2 | 3.5% | 19 | 33.3% | 6 | 10.5% | 57 | 100.0% |
| **Total** | | 34 | 12.1% | 79 | 28.0% | 15 | 5.3% | 102 | 36.2% | 52 | 18.4% | 282 | 100.0% |
| **6. HR manager consulted from the outset at the development of organizational strategy** | Food | 19 | 27.5% | 11 | 15.9% | 4 | 5.8% | 23 | 33.3% | 12 | 17.4% | 69 | 100.0% |
|  | Textile | 1 | 1.3% | 16 | 20.3% | 4 | 5.1% | 27 | 34.2% | 31 | 39.2% | 79 | 100.0% |
|  | Metal | 4 | 5.2% | 38 | 49.4% | 11 | 14.3% | 12 | 15.6% | 12 | 15.6% | 77 | 100.0% |
|  | chemical | 9 | 15.8% | 19 | 33.3% | 1 | 1.8% | 12 | 21.1% | 16 | 28.1% | 57 | 100.0% |
| **Total** | | 33 | 11.7% | 84 | 29.8% | 20 | 7.1% | 74 | 26.2% | 71 | 25.2 | 282 | 100.0% |
| **7. Existence of a written HR strategy** | Food | 10 | 14.5% | 28 | 40.6% | 3 | 4.3% | 22 | 31.9% | 6 | 8.7% | 69 | 100.0% |
|  | Textile | 11 | 13.9% | 39 | 45.6% | 1 | 1.3% | 24 | 30.4% | 7 | 8.9% | 79 | 100.0% |
|  | Metal | 23 | 29.9% | 23 | 29.9% | 5 | 6.5% | 21 | 27.3% | 5 | 6.5% | 77 | 100.0% |
|  | chemical | 11 | 19.3% | 21 | 36.8% | 1 | 1.8% | 16 | 28.1% | 8 | 14.0% | 57 | 100.0% |
| **Total** | | 55 | 19.5% | 108 | 38.3% | 10 | 3.5% | 83 | 29.4% | 26 | 9.2% | 282 | 100.0% |
| **8. HR strategy is translated into a clear set of workable programs** | Food | 21 | 30.4% | 21 | 30.4% | 0 | 0.0% | 17 | 24.6% | 10 | 14.5% | 69 | 100.0% |
|  | Textile | 24 | 30.4% | 34 | 43.0% | 4 | 5.1% | 12 | 15.2% | 5 | 6.3% | 79 | 100.0% |
|  | Metal | 21 | 27.3% | 30 | 39.0% | 3 | 3.9% | 20 | 26.0% | 3 | 3.9% | 77 | 100.0% |
|  | chemical | 17 | 29.8% | 19 | 33.3% | 3 | 5.3% | 13 | 22.8% | 5 | 8.8% | 57 | 100.0% |
| **Total** | | 83 | 29.4% | 104 | 36.9% | 10 | 3.5% | 62 | 22.0% | 23 | 8.2% | 282 | 100.0% |
